# Improved Response to Disasters and Outbreaks by Tracking Population Movements with Mobile Phone Network Data: A Post-Earthquake Geospatial Study in Haiti

**DOI:** 10.1371/journal.pmed.1001083

**Published:** 2011-08-30

**Authors:** Linus Bengtsson, Xin Lu, Anna Thorson, Richard Garfield, Johan von Schreeb

**Affiliations:** 1 Department of Public Health Sciences, Karolinska Institutet, Stockholm, Sweden; 2 Department of Sociology, Stockholm University, Stockholm, Sweden; 3 Schools of Nursing and Public Health, Columbia University, New York City, United States of America; University of Oxford, United Kingdom

## Abstract

**Background:**

Population movements following disasters can cause important increases in morbidity and mortality. Without knowledge of the locations of affected people, relief assistance is compromised. No rapid and accurate method exists to track population movements after disasters. We used position data of subscriber identity module (SIM) cards from the largest mobile phone company in Haiti (Digicel) to estimate the magnitude and trends of population movements following the Haiti 2010 earthquake and cholera outbreak.

**Methods and Findings:**

Geographic positions of SIM cards were determined by the location of the mobile phone tower through which each SIM card connects when calling. We followed daily positions of SIM cards 42 days before the earthquake and 158 days after. To exclude inactivated SIM cards, we included only the 1.9 million SIM cards that made at least one call both pre-earthquake and during the last month of study. In Port-au-Prince there were 3.2 persons per included SIM card. We used this ratio to extrapolate from the number of moving SIM cards to the number of moving persons. Cholera outbreak analyses covered 8 days and tracked 138,560 SIM cards.

An estimated 630,000 persons (197,484 Digicel SIM cards), present in Port-au-Prince on the day of the earthquake, had left 19 days post-earthquake. Estimated net outflow of people (outflow minus inflow) corresponded to 20% of the Port-au-Prince pre-earthquake population. Geographic distribution of population movements from Port-au-Prince corresponded well with results from a large retrospective, population-based UN survey. To demonstrate feasibility of rapid estimates and to identify areas at potentially increased risk of outbreaks, we produced reports on SIM card movements from a cholera outbreak area at its immediate onset and within 12 hours of receiving data.

**Conclusions:**

Results suggest that estimates of population movements during disasters and outbreaks can be delivered rapidly and with potentially high validity in areas with high mobile phone use.

*Please see later in the article for the Editors' Summary*

## Abstract 2

Linus Bengtsson and colleagues examine the use of mobile phone positioning data to monitor population movements during disasters and outbreaks, finding that reports on population movements can be generated within twelve hours of receiving data.

## Editors' Summary

**Background:**

Every year, millions of people are affected by disasters—sudden calamitous events that disrupt communities and cause major human, material, economic, and environmental losses. Disasters can be natural (for example, earthquakes and infectious disease outbreaks) or man-made (for example, terrorist attacks and industrial accidents). Whenever a disaster strikes, governments, international bodies, and humanitarian agencies swing into action to help the affected population by providing food, water, shelter, and medical assistance. Within days of the earthquake that struck Haiti on January 12, 2010, for instance, many governments pledged large sums of money to help the Haitians, and humanitarian agencies such as Oxfam and the International Federation of Red Cross and Red Crescent Societies sent tons of food and hundreds of personnel into the country. And when a cholera outbreak began in Haiti in October 2010, the world responded by sending further assistance.

**Why Was This Study Done?:**

An instinctive response to any disaster is to flee the affected area, but such population movements after a disaster can increase the loss of human life by complicating the provision of relief assistance, the assessment of needs, and infectious disease surveillance. Unfortunately, there are no rapid or accurate methods available to track population movements after disasters. Relief coordinators currently rely on slow, potentially biased methods such as eye witness accounts and aerial images of shelters to track population movements. In this geospatial analysis, the researchers investigate whether position data from mobile phone SIMs (subscriber identity modules) can be used to estimate the magnitude and trends of population movements by retrospectively following the positions of SIMs in Haiti before and after the earthquake and tracking SIMs during the first few days of the cholera outbreak. Every time a SIM makes a call, the mobile phone network database records which mobile phone tower connected the call. Thus, the database can provide a geographic position for each mobile phone caller.

**What Did the Researchers Do and Find?:**

The researchers obtained anonymized data on the position of 1.9 million SIMs in Haiti from 42 days before the earthquake to 158 days afterwards. Nearly 200,000 SIMs that were present in Haiti's capital Port-au-Prince when the earthquake struck had left 19 days post-earthquake. Just under a third of Port-au-Prince's inhabitants were mobile phone subscribers at the time of the earthquake, so this movement of SIMs equates to the movement of about 630,000 people. Notably, although the SIM-based estimates of numbers leaving Port-au-Prince matched the estimates reported by the Haitian National Civil Protection Agency (NPCA), which were largely based on counting ship and bus movements and which were used during the relief operation, the estimated geographical distribution of displaced people reported by the NPCA was very different to that obtained by analyzing SIM movements. By contrast, the geographical distribution of the population obtained from SIM movements closely matched that reported by a retrospective United Nations Population Fund household survey. Finally, to demonstrate the feasibility of producing rapid estimates of population movements during disasters, the researchers tracked nearly 140,000 SIMs during the first 8 days of the Haitian cholera outbreak and showed that they could distribute analyses of SIM movements within 12 hours of receiving data from the mobile phone company.

**What Do These Findings Mean?:**

These findings suggest that estimates of population movements during disasters and infectious disease outbreaks can be delivered rapidly and accurately in areas of high mobile use by analyzing mobile phone data. 86% of the world's population now has mobile phone network coverage and, in 2009, there were already 3.2 billion mobile phone subscriptions in the developing world, which has a population of 5.5 billion people. Thus, this tracking method could be useful in many parts of the world, including those particularly vulnerable to disasters. However, because mobile phone use varies between sections of society, because some areas have a low density of mobile phone towers, and because disasters can destroy these towers, this approach may not be effective in all disasters. The researchers recommend, therefore, that the use of mobile phone data for tracking population movements is evaluated further and that relationships are built up with mobile phone companies to ensure rapid implementation of the approach after future disasters.

**Additional Information:**

Please access these Web sites via the online version of this summary at http://dx.doi.org/10.1371/journal.pmed.1001083.

- Internal Displacement Monitoring Centre provides information on population displacement during natural disasters
- The International Federation of Red Cross and Red Crescent Societies provides information in several languages about disaster management and about the 2010 earthquake in Haiti
- Oxfam also has information on conflicts and natural disasters and the Haiti earthquake and cholera outbreak (in several languages)
- EM-DAT, the International Disaster Database contains essential core information on 18,000 mass disasters in the world from 1990 until the present

## Introduction

Sudden onset natural disasters displaced an estimated 36 million people in 2008 [1]. Rapid, uncontrolled population movements often lead to important increases in morbidity and mortality [2],[3]. During outbreaks of cholera in displaced populations, mortality is reported to have increased more than 50-fold compared to baseline rates during stable conditions [4]. Population movements following natural disasters can severely complicate provision of relief assistance, needs assessments, and infectious disease surveillance.

There is no method to rapidly and accurately follow population movements after disasters and disease outbreaks, and estimates are often little more than educated guesses. Relief coordinators currently rely on eyewitness accounts, manual counting of people, registration of people in camps, or satellite or aerial images of shelters or changes in vegetation [5]–[7]. These approaches are often subject to heavy bias or are too slow to suit rapidly changing conditions.

New technologies hold promise to improve the quality of information on population displacement [8]. A total of 86% of the world's population lives under mobile cellular network coverage [9]. In 2009 the number of mobile phone subscriptions in the developing world reached 3.2 billion, out of a total population of 5.5 billion persons (3.9 billion persons of age 15 y and above) [10],[11]. Mobile phones have been used as communication devices during disasters, letting professionals or laypersons relay information to coordinating units [12],[13]. However, in addition to enabling communication, mobile phone networks routinely register data that can be used to track the location of all active mobile phones. This data source has been used to analyse mobility patterns under stable societal conditions [14]–[16], but not during major societal disruptions such as a natural disaster or a large infectious disease outbreak under unstable conditions.

During the first weeks after the Haiti earthquake on January 12, 2010 there were reports of large population movements out of the severely affected capital, Port-au-Prince (PaP) [17]. In October 2010 a cholera outbreak also occurred in Haiti [18]. Rumours about large population movements out of the outbreak area circulated and it was imperative to identify high-risk areas for the emergence of new outbreaks.

Several of the research team members worked in Haiti to plan and deliver relief activities following the earthquake. The lack of reliable data on population movement made coordination and relief prioritization difficult, and it is within this context that this study was launched. The aims of this study were to: (1) estimate the magnitude and trends of population movements from PaP following the January 2010 earthquake using mobile phone network data; (2) compare the results with other data sources; and (3) to assess the feasibility of using mobile phone network data to rapidly track population movements during the October 2010 cholera outbreak in Haiti.

## Materials and Methods

### Ethics Statement

The study was approved by the Regional Ethical Review Board, Stockholm, Sweden.

### Study Rationale

All mobile phones have a subscriber identity module, commonly known as a SIM card (henceforward SIM). A SIM communicates with a mobile phone network through mobile phone towers. Every time a SIM calls, the mobile phone network database records which tower connects the call. This database allows each SIM card's position to be followed over time with the accuracy of the mobile phone towers' coverage areas. Coverage areas vary from approximately 1–100 km^2^ [14]–[16].

### Data Collection

There are an estimated 3.5 million mobile phone subscribers among Haiti's 10 million inhabitants [19]. Haiti has two GSM mobile phone network operators. We have not found evidence of differences in the characteristics of the companies' subscriber populations. The largest company, Digicel, with 2.2 million subscribers [20] and a network that covers 90% of the inhabited areas [21], provided us with anonymous data on all SIMs that made at least one call during the study periods. The data contained, for each call, the location of the mobile phone tower used. Period one (December 1, 2009 to June 18, 2010) started 6 wk before the earthquake and lasted until approximately 5 mo after the earthquake. Period two (October 15 to October 23, 2010) covered the early phase of the cholera outbreak. The mobile phone network faced reduced capacity immediately after the earthquake but was functioning within a few days.

The data contained records of 2.8 million SIMs and 282 million registered calling locations. Data for period one included the position of the mobile phone tower used by each SIM at the time of its first call each day. Data for period two included the position of the mobile phone tower used by each SIM at the time of each call. Digicel also shared maps of the network's towers and coverage areas with the research team. We retrieved data on the borders of Haiti's administrative divisions [22] and official estimated population sizes for 2009 [23].

### Study Population and Inclusion Criteria

For study period one, we included all SIMs that made at least one call during the 6-wk period prior to the earthquake (December 1, 2009 to January 12, 2010) and that also made at least one call during the last month of the study period (May 18 to June 18, 2010). The former excluded the large numbers of relief workers arriving after the earthquake, while the latter excluded SIMs that were lost or destroyed in or after the earthquake. A total of 1.9 million unique SIMs fulfilled the inclusion criteria. For study period two (cholera outbreak analyses) we included all SIMs that made at least one call within the outbreak area during study period two.

### Definitions and Assumptions

We defined the PaP metropolitan area as the communal sections lying within the urban sprawl of PaP. We defined the position of a SIM at any given date by its latest registered position. On average, 67% of the SIMs called more often than every second day and 80% called more often than every fourth day. On the day of the earthquake there were 809,573 SIMs in PaP that were included in the study, covering a population of 2.6 million [23]. The ratio of the SIMs to the population was 31%. We assumed that movements of PaP SIMs were representative of movements of the general PaP population. Hence, in our estimates each movement of a SIM that was present in PaP during study period one represented 3.2 persons (1/0.31). However, mobile phone use is low (or lower than average) in several population groups including children, the elderly, the poorest, and women [24]. If these groups have substantially different movement patterns than groups with high mobile phone use, results would be biased. To address these concerns we make a comparison between SIM and population-based data in the results and we discuss the assumption of representatively in detail in the discussion. In the cholera outbreak analyses, we defined the cholera outbreak area on the basis of the information on the location of cases that was available at the immediate onset of the epidemic. This area included four communes and three communal sections around the city of St Marc. The ratio of SIMs to the population in this area was 21% (138,560 SIMs, 648,717 people). See also STROBE statement (Text S1).

### Analyses

For each SIM we produced a list of its location on each day during the study periods. This database was then used to summarize the number of SIMs located in the respective administrative areas in Haiti (departments, communes, and communal sections) during study period one and two. Data were stored and managed in MS SQL server 2005. Analyses were performed in Microsoft Visual C# 2008, Matlab R2008c, and ArcGIS 9.2.

### Comparison of Results with Other Estimates of Population Movements

We compared our results with estimates from the Haitian National Civil Protection Agency that were widely distributed and used by relief agencies during the first months following the earthquake. These estimates were to a large extent based on individual counting of buses and ships leaving the area [25].

We also compared our results to a retrospective population-based household survey performed in PaP by United Nations Population Fund (UNFPA) [26]. The survey included questions on individual post-earthquake movements by PaP inhabitants from January 12 to September 10, 2010. The study included 2,500 households with an average household size of 4.9 persons. Results from the survey included point frequencies for the following questions (translated from French): (1) “Did you leave the metropolitan area after January 12 (earthquake day) even if it was for a short time” and (2) “to what department (province) did you go?” The first question (“leave for a short period of time”) is sensitive to individual interpretation. For comparison we hence produced one geographic distribution for SIMs that spent at least 2 d outside PaP after the earthquake and another distribution for those that spent at least 7 d outside PaP after the earthquake. In order to match the UNFPA survey as closely as possible, we included all SIMs that were present in PaP on the day of the earthquake and that had returned to PaP before the end of study period one. We defined a period of stay outside PaP as the period between two consecutive calls from outside PaP.

## Results

### Magnitude of Population Movements

**Figure 1:**
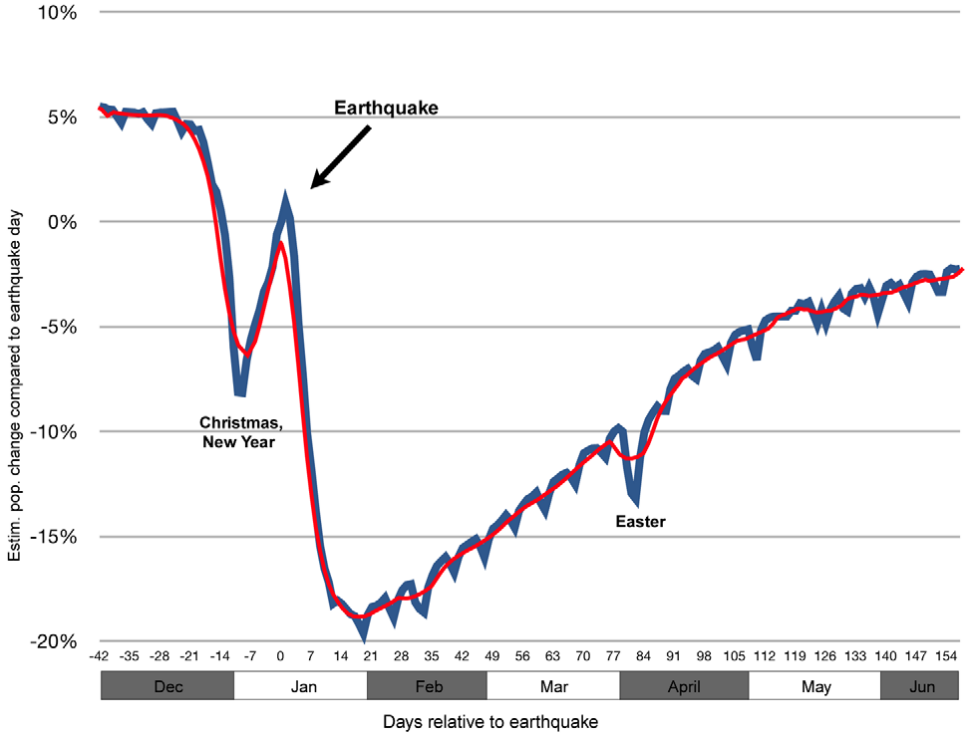
Estimated net changes of the PaP population compared to the population of PaP on the earthquake day (7-d moving average shown in red). The earthquake caused a sharp net outflow from PaP. The PaP population then gradually grew until the end of the study period. The wavy shape of the curve is caused by net inflows during working days and corresponding net outflows during weekends. Sharp but temporary increases and decreases in the PaP population occurred during major holidays.

Population movements following the earthquake were very large. The PaP population size is estimated to have reached its lowest level 19 d after the earthquake (January 31), after which it started to recover (Figure 1). At this time an estimated 630,000 people (197,484 SIMs) who were present in PaP on the earthquake day had left PaP and not yet returned. Concurrently, an estimated 120,000 persons (38,729 SIMs) who were outside PaP on the earthquake day had moved into PaP at 19 d post-earthquake. The net outflow from PaP during this early period was thus 510,000 persons (158,755 SIMs) or 20% of the PaP pre-earthquake population.

### Distribution of the Moving PaP Population

**Figure 2:**
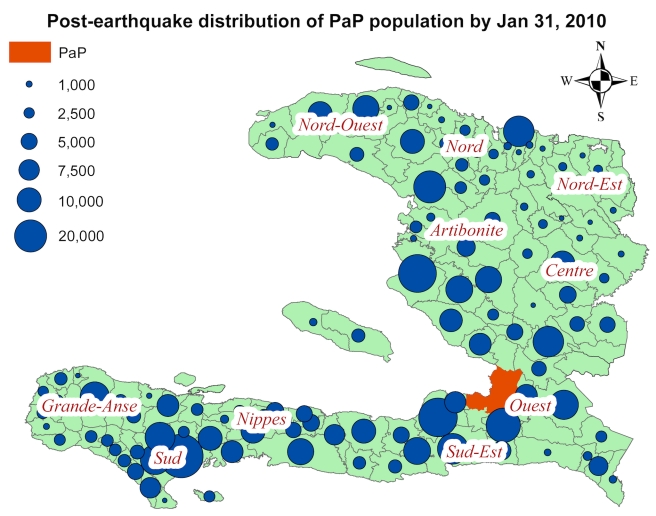
Estimated distribution of persons who were in PaP on the day of the earthquake but outside PaP 19 d after the earthquake. Circles are shown for communes that received at least 500 persons.

People who left PaP after the earthquake moved to almost all parts of the country. However, the distribution between different communes was highly uneven. We identified the major recipient communes to be: Les Cayes, Leogane, and Saint-Marc, at the peak of net out migration from PaP 19 d after the earthquake (Figure 2). These communes received an estimated 28,000, 23,000, and 22,000 persons from PaP, respectively.

### Comparisons with Early Estimates Used during the Relief Operation

**Figure 3:**
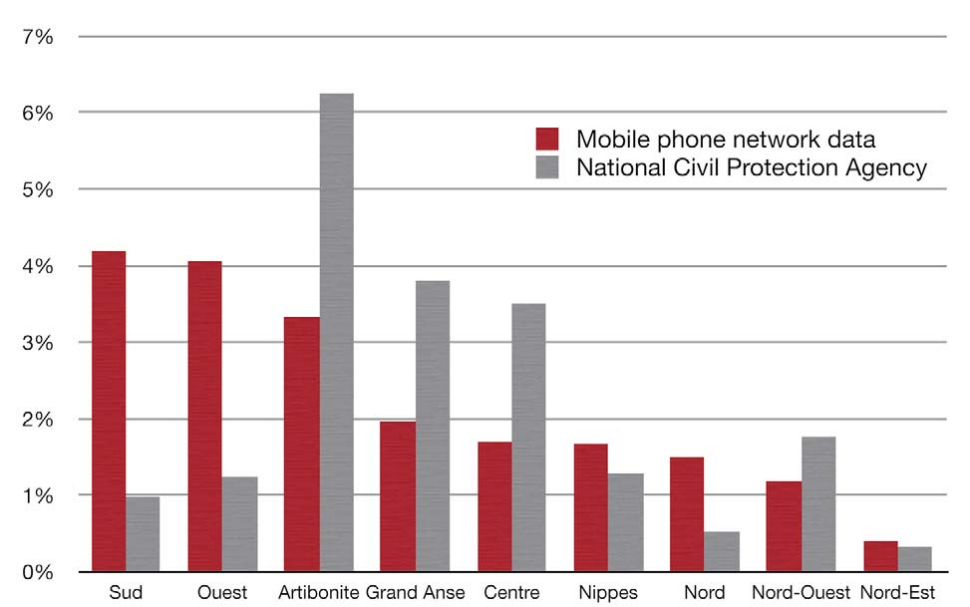
Estimates of the proportion of PaP persons who had left PaP by February 17, 2010, divided per province. Red, mobile phone network data; grey, National Civil Protection Agency (NCPA) estimates used by the Office for the Coordination of Humanitarian Affairs (OCHA) and other relief agencies.

The National Civil Protection Agency (NCPA) estimated that 511,405 persons had left the capital on the February 17 [27], compared to our estimate of 580,000 individuals for the same date. However the estimated distribution of these persons across the country was very different (Figure 3). NCPA's estimate of the numbers of individuals who had moved to the departments Sud and Ouest was even lower than the number of individual SIMs that had moved to these two departments.

### Comparison with Retrospective Population-Based Survey Data

**Figure 4:**
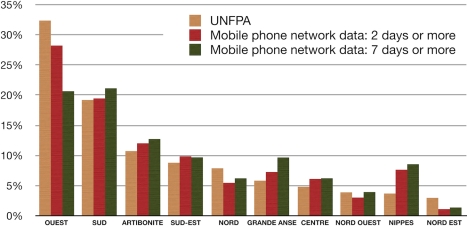
Distribution of PaP persons who left and returned to the city following the earthquake. UNFPA household survey (beige) and population estimates based on SIMs that spent a minimum of 2 and 7 d outside PaP respectively (red and green).

Our estimates of the geographical distributions across Haiti were similar to the estimates derived from the retrospective UNFPA study [26], which included a representative sample of 2,500 households in PaP and among which 2,921 persons had left PaP following the earthquake (Figure 4).

### Cholera Outbreak Response: Feasibility of Producing Rapid Data on Movements

**Figure 5:**
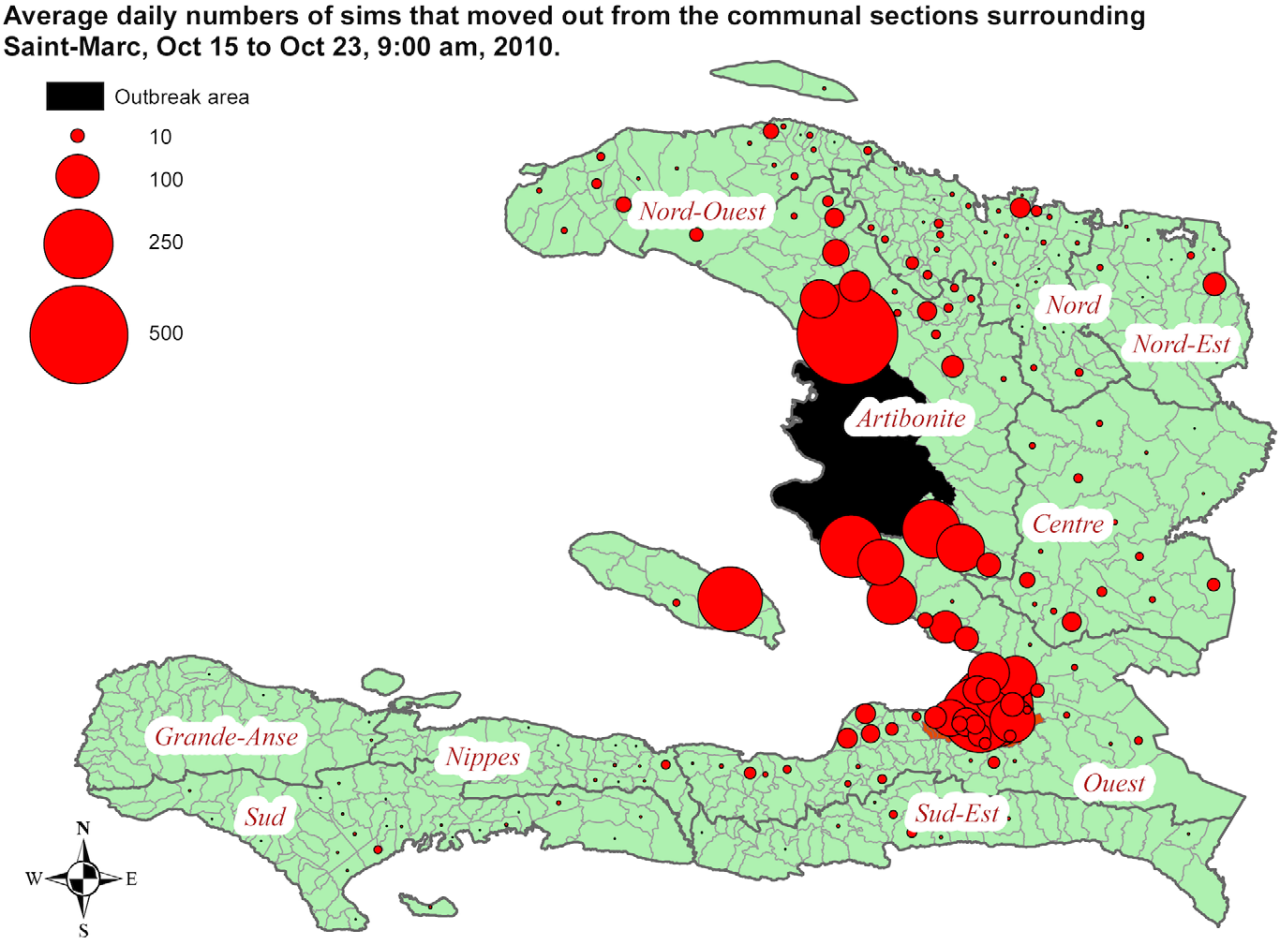
Average daily numbers of SIMs moving out of the cholera outbreak area. October 15 to October 23, 2010, divided per communal section of destination. The data were disseminated to relief agencies at the outset of the outbreak (October 24, 2010).

In order to provide information on areas at potentially increased risk of new outbreaks as well as on areas with higher likelihood of receiving cases in need of treatment, we disseminated data on SIMs moving out of the cholera outbreak area. These analyses were distributed at the immediate onset of the epidemic and within 12 h after receiving data from Digicel. We found that a daily average of 3,676 SIMs (2.7% of SIMs in the area) left the outbreak area during the 8-d study period. These SIMs moved to all provinces in the country but were heavily concentrated in communal sections in PaP, areas surrounding the outbreak area, and urban centres north and northeast of the outbreak area. The Southwest part of the country received very few SIMs (Figure 5). Despite significant news coverage about the outbreak, the number of SIMs leaving the outbreak area did not increase during the period (as compared to the preceding 1 mo).

## Discussion

We found that an estimated 630,000 people left PaP during the first 19 d following the earthquake. The geographic distribution of people who moved out of PaP was highly nonuniform, and was similar to estimates from a large retrospective population-based UN survey but very different from the official government estimates which were widely used during the relief operations. We additionally illustrate that it was feasible to produce and disseminate data on movements of SIM cards from an area with an infectious disease outbreak within hours of receiving the data. These results suggest that the speed and accuracy of estimates of population movements during disasters and infectious disease outbreaks may be revolutionized in areas with high mobile phone coverage.

There has been no method available to provide timely and accurate estimates of population movements following many disasters. Large-scale surveys and censuses can give detailed information on people's movement history but are not feasible to implement for monitoring purposes during the acute phase of a disaster. Eyewitness reports may carry severe bias when referring to population movements occurring over time and across areas.

There are several limitations to this study. Mobile phone use is low in several population groups, including children, the elderly, the poorest, and women [24]. If these groups have substantially different movement patterns than groups with high mobile phone use, results will be biased. However, the proportional geographic distribution of people moving away from PaP agreed well with the results of a large retrospective population-based survey by UNFPA. The similarities can be due to a widespread use of mobile phones, that nonmobile phone users, e.g., children and the elderly, moved together with mobile phone users, and also that users and nonusers of mobile phones had similar movement patterns. The UNFPA study was however not implemented for validation purposes and future population-based studies in other settings are needed. SIM movements will however unequivocally provide a valuable lower bound on population movements if ownership of multiple SIMs from the same company is uncommon. In the present study the SIMs would then have tracked a minimum of 31% of the PaP population (809,000 persons). If for example children and the elderly, who do not use mobile phones, accompany people who do, the number of persons who are directly tracked by the method would be substantially higher.

The method may be less suitable in areas where mobile phone use and mobile radio coverage is low. Geographic localisation is less precise in areas with low tower density. When relying on call data, locations of infrequent callers are updated less often than those of frequent callers. As well, in some countries, companies put large amounts of airtime on new SIMs to attract new customers. This can lead some customers to keep one stable SIM for receiving calls while new SIMs are continuously bought and used for making calls. This was not the case in Haiti but needs to be taken into account in other contexts. Additionally, there are settings where many people have one SIM card from each company, which needs to be taken into account if data from more than one company is analyzed.

Mobile phone networks are relatively resilient to external shocks. However, major disasters can affect power supply, destroy towers, and cause a complete loss of functionality. Limited possibilities for people to charge their mobile phones can cause bias and might have done so in this study. In Haiti, power cuts were common before the earthquake and existing electrical generators seem to have supplied considerable charging capacity. Other partially compensating factors for power cuts include long stand-by times of many simple inexpensive phones as well as the habit of people in places with insufficient power supply to routinely turn off phones to conserve battery.

Although we did not have access to other data types, network registries contain more detailed data that may be analyzed. Such data include for example all calls (as compared to once daily in the present dataset), text messages and data downloads (but not the content of these), data on expenditure and the size of prepaid refills, as well as SIM location data that are regularly registered by the system without SIMs making calls. Some of these data are difficult to rapidly retrieve and some companies regularly erase certain data types. Discussion with the specific company in each case is important. Data should always be made anonymous before analysts access it, as was done in this study.

With software development and in cooperation with network operators, the described approach can provide data in close to real time. Information on postdisaster population distributions can potentially enable improved distribution of water, food, shelter, and sanitation. Needs assessment surveys can potentially be improved through increased validity of population estimates. These denominator estimates would be important both when constructing sampling frames for needs assessment surveys and when generalizing survey data to overall population needs. For example, survey data indicating that 50% of the people in an area needs shelter are tremendously more informative if the area has an accurately estimated population size. Daily estimated changes in the number of displaced persons can be generated for specified areas, which can signal important on-going developments. Estimates of mortality can potentially be derived from the number and geographic distribution of nonresponding SIMs. Estimation of buried but alive persons following an earthquake is another potential area for development. Network data can provide even richer information when combined with information on the ground and potentially also with data from mobile phone surveys [13].

Our approach may also be useful in nondisaster contexts. Human mobility is extremely important for the spread of communicable diseases [28],[29], and early containment of epidemic outbreaks is often a key factor in preventing spread [30]. Rapid data on population movements can potentially inform outbreak preparedness and response for infectious diseases. In addition, the coarse data on diagnosed cholera cases that are presently available [31] show interesting similarities to the mobile phone network data (Figure 5). More detailed comparisons will be fruitful to perform if data on the location of diagnosed cases become available. Text messaging that targets specific areas is another potentially valuable use of mobile phone network data. If these refer to advice relating to population movements (e.g., advice regarding evacuation or the localisation of relief supplies), results could be directly evaluated with the use of network data. During the cholera outbreak, Digicel sent, on the basis of the presented analyses, text messages with health information to all mobile phones that passed through the outbreak area. This project was then expanded to include an automatic voice dialler and a total of seven text messages per mobile phone subscriber.

### Conclusions

We found that routinely collected data on the movements of all active SIM cards in a disaster-affected nation could, with potentially high validity, be used to provide estimates of the magnitude, distribution, and trends in population displacement. With pre-earthquake census data, the method could also provide estimates on area-specific population sizes, which could lead to important improvements in the allocation of relief supplies and the quality of needs assessment surveys. Further, we found that the method was feasible to use for close to real-time monitoring of population movements during an infectious disease outbreak. We recommend establishing relations with mobile phone operators prior to emergencies as well as implementing and further evaluating the method during future disasters.

## Supporting Information

Text S1 **STROBE statement.**
(DOC)

## Abbreviations

PaP: Port-au-Prince
SIM: subscriber identity module
UNFPA: United Nations Population Fund
